# Supplementary material for: HOX13-dependent chromatin accessibility underlies the transition towards the digit development program
Source: Nat Commun. 2020 May 19;11:2491. doi: 10.1038/s41467-020-16317-2 (PMC7237422; doi:10.1038/s41467-020-16317-2)
Supplement: Supplementary file 1 — Supplementary Information [file 41467_2020_16317_MOESM1_ESM.pdf]

Supplementary Information

HOX13-dependent chromatin accessibility underlies the transition towards the digit development program

Desanlis et al.

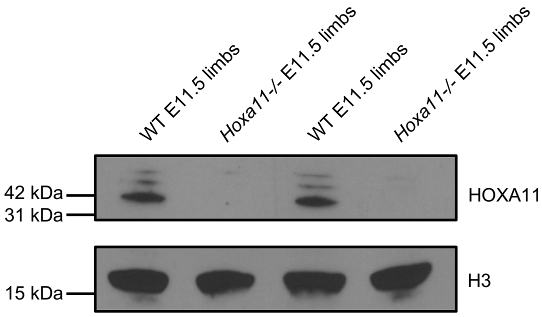

Supplementary Figure 1: Validation of HOXA11 antibody specificity

Western blot of nuclear extracts from E11.5 wild type and *Hoxa11*$^{-/-}$ limb buds. Two replicates are shown. Antibody against HOXA11 (top panel) and Histone H3 (bottom panel) were used. The samples for both western blots derive from the same experiment and blots were processed in parallel.

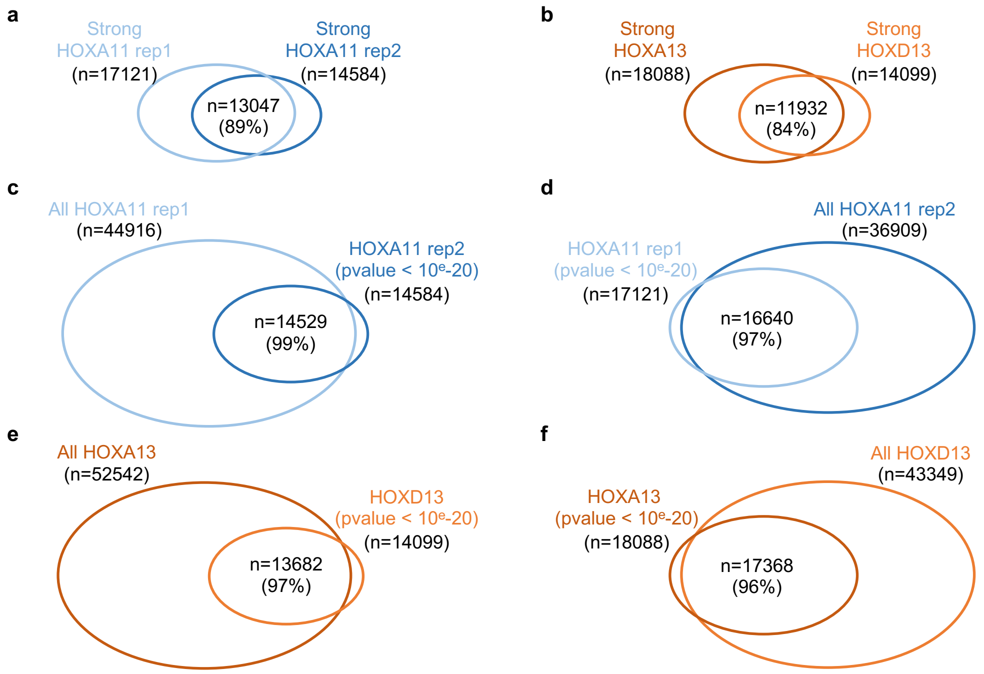

g

	HOXA13		HOXD13		HOXA11 rep1		HOXA11 rep2	
	All	Strong	All	Strong	All	Strong	All	Strong
HOXA11 specific: n=2128	-	-	-	-	+	+	+	+
Common HOXA11-HOX13 n=6967	+	+	+	+	+	+	+	+
HOX13 specific: n=2130	+	+	+	+	-	-	-	-

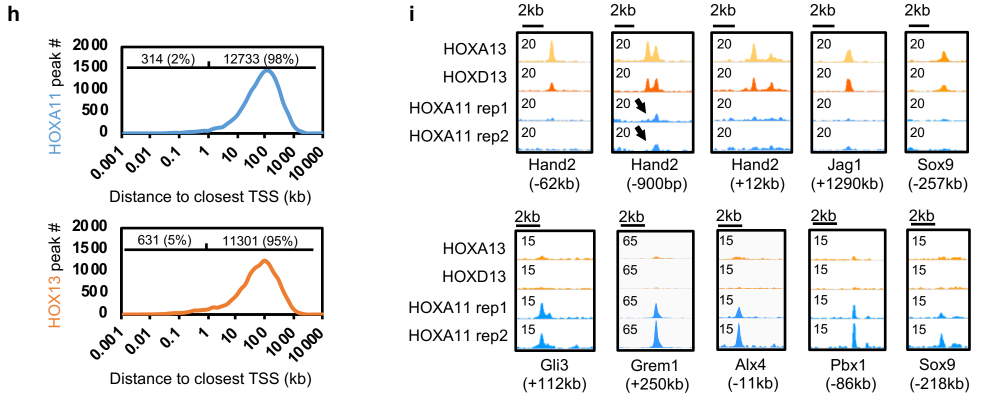

Supplementary Figure 2: Comparison between HOXA11 and HOXA/D13 binding

(**a-f**) Venn diagrams showing the number of peak overlaps between the indicated ChIP-seq datasets. Strong peaks refer to peaks with a pvalue $<10^{-20}$ and all peaks refer to peaks with an associated pvalue of $<10^{-5}$ (Poisson distribution p-value based on lambda and corrected for multiple comparison using the Benjamini-Hochberg correction computed by MACS2). Two peaks were considered overlapping whenever the peak window of the two peaks showed any overlap. (**g**) Parameters used to define HOXA11 specific, Common peaks and HOX13-specific peaks in Fig. 1d. (**h**) Distance of the HOXA11 (blue) and HOXA13 (orange) bound loci to the closest TSS of all HOXA11 and HOXA13 peaks (p-value < 10^{-5}, Poisson distribution p-value based on lambda computed and corrected for multiple comparison using the Benjamini-Hochberg correction by MACS2) in E11.5 forelimb buds. (**i**) Genome browser view (IGV) of a few example loci with HOX13-specific (top) and HOXA11-specific (bottom) peaks. Source data are provided as a Source Data file

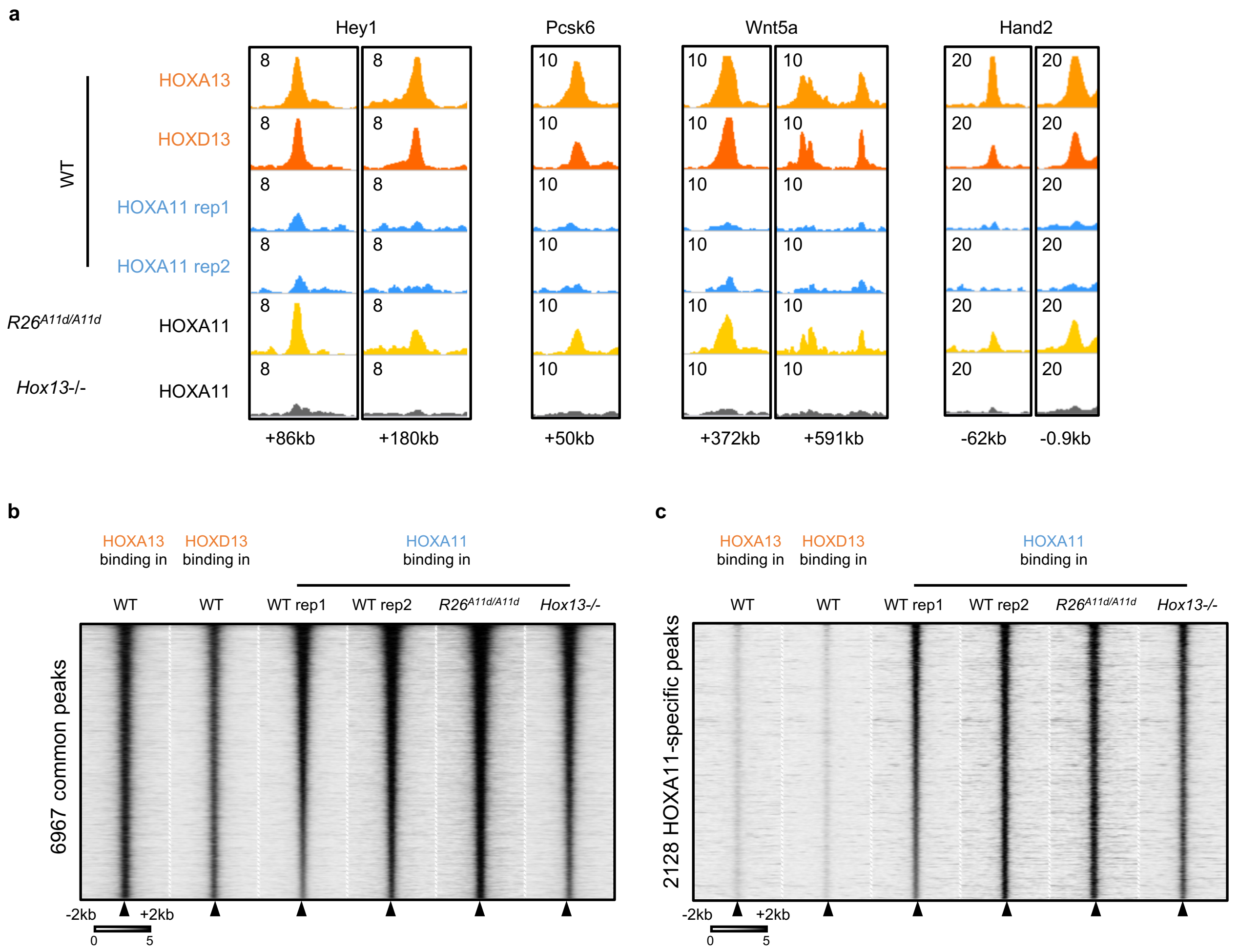

Supplementary Figure 3: HOXA11 ectopic binding after distal HOXA11 expression

(a) Genome browser view (IGV) of a few example loci with ectopic binding of HOXA11 only when co-expressed distally with HOX13. (**b**) Heatmaps showing a 4kb window of ChIP-seq read density for HOXA13/HOXD13 in wild type (left), HOXA11 in wild type (middle), *R26*$^{A11d/A11d}$ and *Hox13*$^{-/-}$ E11.5 forelimb buds (right) at common peaks with a p-value $<10^{-20}$. Peaks are ranked based on p-value of HOXA13 binding (Poisson distribution p-value based on lambda and corrected for multiple comparison using the Benjamini-Hochberg correction computed by MACS2). (**c**) Heatmaps showing a 4kb window of ChIP-seq read density for HOXA13/HOXD13 in wild type (left), HOXA11 in wild type (middle), *R26*$^{A11d/A11d}$ and *Hox13*$^{-/-}$ E11.5 forelimb buds (right) at HOXA11 specific peaks with a p-value $<10^{-20}$.Peaks are ranked based on p-value of HOXA13 binding (Poisson distribution p-value based on lambda and corrected for multiple comparison using the Benjamini-Hochberg correction computed by MACS2). Color scale indicates reads per million reads (RPM). Source data are provided as a Source Data file.

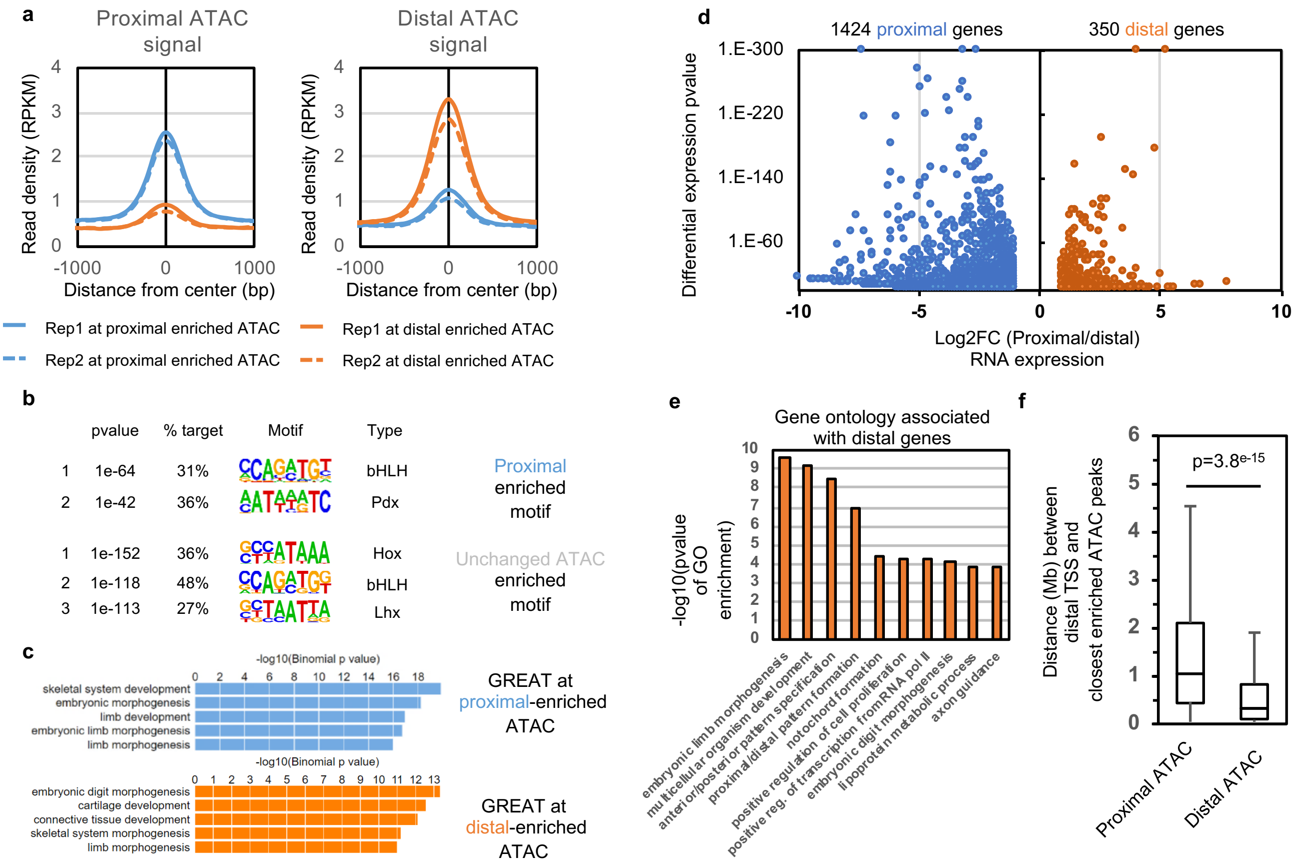

Supplementary Figure 4: Characterization of proximo-distal E11.5 limbs open chromatin landscapes

(a) Average profile showing proximal and distal ATAC-seq signals at proximal enriched (left) and distal enriched (right) ATAC-seq peaks. **(b)** *de novo* motif analyses (HOMER) at proximal enriched (top) and at unchanged (bottom) ATAC peaks using the whole open region as input rather than a fixed window. **(c)** GREAT analysis showing the enriched GO terms for biological processes for genes associated with proximal (top, blue) and distal (bottom orange) ATAC-seq peaks. **(d)** Volcano Plot showing the log2 fold change of proximal over distal RNA expression (on the X axis) and the adjusted p-value computed by DESEQ2 differential expression analysis (on the Y axis). Proximally enriched genes are shown in blue while distally enriched genes are in orange. **(e)** Gene ontology associated with distally enriched genes. **(f)** Boxplot showing the distance between the TSS of distal enriched genes and the closest proximal- (left) or distal- (right) enriched ATAC-seq peak. Center lines show medians; box limits indicate the twenty fifth and seventy-fifth percentiles; whiskers extend to 1.5 times the interquartile range from the twenty-fifth to seventy-fifth percentiles. p-value was calculated using unpaired two-tailed student t-test. Source data are provided as a Source Data file.

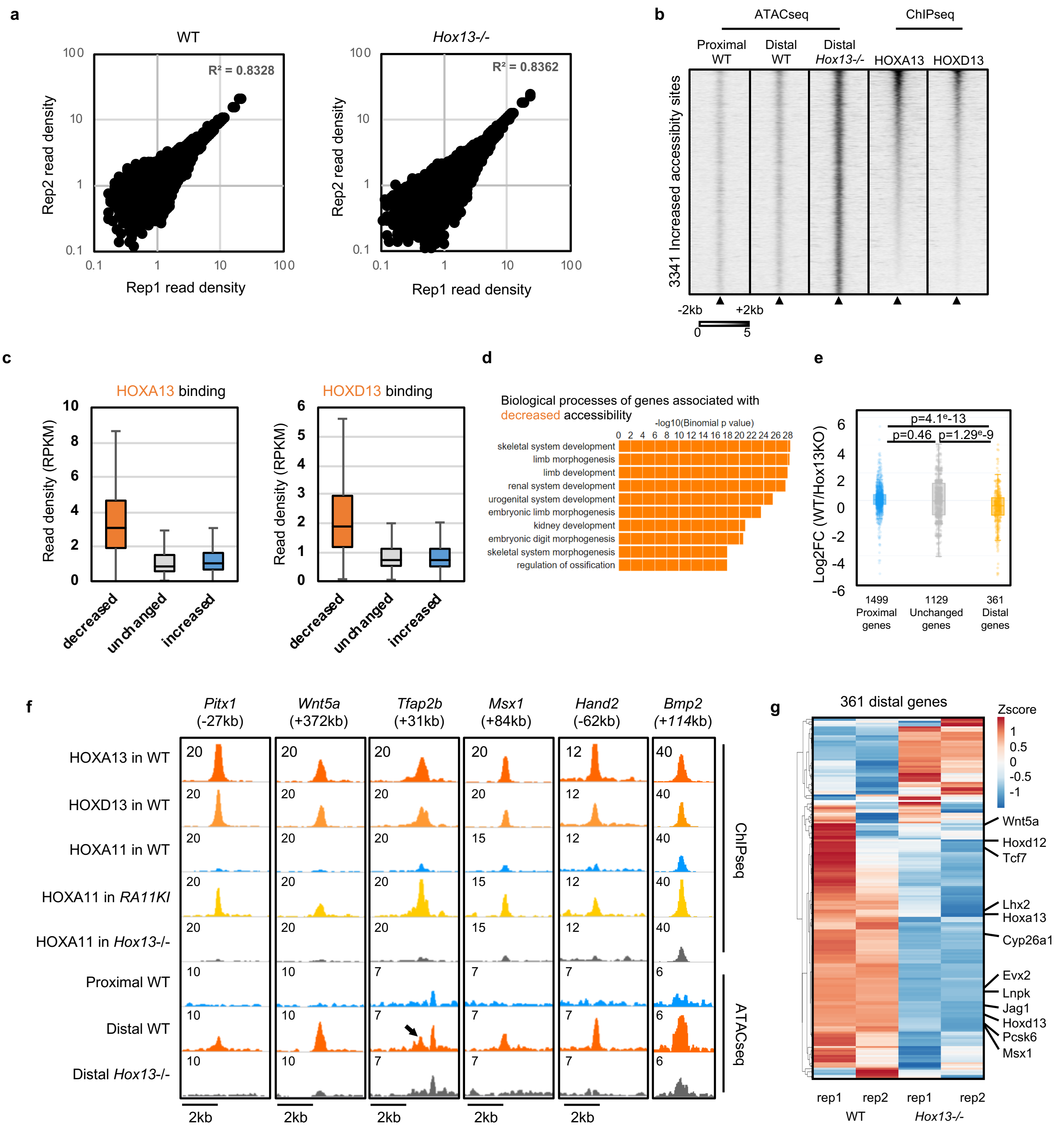
a
WT
Hox13-/-
R² = 0.8328
R² = 0.8362
Rep2 read density
Rep1 read density
b
ATACseq
ChIPseq
Proximal WT
Distal WT
Distal Hox13-/-
HOXA13
HOXD13
3341 Increased accessibity sites
-2kb
+2kb
c
HOXA13 binding
HOXD13 binding
Read density (RPKM)
decreased
unchanged
increased
d
Biological processes of genes associated with decreased accessibility
-log10(Binomial p value)
skeletal system development
limb morphogenesis
limb development
renal system development
urogenital system development
embryonic limb morphogenesis
kidney development
embryonic digit morphogenesis
skeletal system morphogenesis
regulation of ossification
e
Log2FC (WT/Hox13KO)
p=4.1e-13
p=0.46
p=1.29e-9
1499 Proximal genes
1129 Unchanged genes
361 Distal genes
f
Pitx1 (-27kb)
Wnt5a (+372kb)
Tfap2b (+31kb)
Msx1 (+84kb)
Hand2 (-62kb)
Bmp2 (+114kb)
HOXA13 in WT
HOXD13 in WT
HOXA11 in WT
HOXA11 in RA11KI
HOXA11 in Hox13-/-
Proximal WT
Distal WT
Distal Hox13-/-
ChIPseq
ATACseq
2kb
g
361 distal genes
Zscore
Wnt5a
Hoxd12
Tcf7
Lhx2
Hoxa13
Cyp26a1
Evx2
Lnpk
Jag1
Hoxd13
Pcsk6
Msx1
rep1
rep2
WT
Hox13-/-

Supplementary Figure 5: Analysis of HOX13-dependent open chromatin landscape

(a) Dispersion plot showing the correlation between the replicates of wild type (left) and *Hox13*-/- (right) ATAC-seq signal (reads per million reads per kb). Pearson correlation is shown. **(b)** Heatmaps showing a 4kb window of ATAC-seq read density for proximal (left) and distal in wild type and *Hox13*$^{-/-}$ (middle), and of ChIP-seq read density for HOXA13 and HOXD13 in wild type (right) E11.5 forelimb buds at increased accessibility specific peaks with a p-value $<10^{-20}$. Peaks are ranked based on p-value of HOXA13 binding (Poisson distribution p-value based on lambda and corrected for multiple comparison using the Benjamini-Hochberg correction computed by MACS2). Color scale indicates reads per million reads (RPM). **(c)** Boxplot showing HOXA13 (left) and HOXD13 (right) binding intensity (reads per million read per kb) in a +/-200bp window surrounding the indicated ATAC peak category. Center lines show medians; box limits indicate the twenty fifth and seventy-fifth percentiles; whiskers extend to 1.5 times the interquartile range from the twenty-fifth to seventy-fifth percentiles. **(d)** GREAT analysis showing the enriched GO terms for biological processes for genes associated with decreased accessibility ATAC-seq peaks. **(e)** Boxplot showing the log2 fold change of WT over *Hox13*$^{-/-}$ differential RNA expression for proximal-enriched, unchanged and distal-enriched genes. Center lines show medians; box limits indicate the twenty fifth and seventy-fifth percentiles; whiskers extend to 1.5 times the interquartile range from the twenty-fifth to seventy-fifth percentiles. pvalue were calculated using unpaired two-tailed student t-test. **(f)** Genome browser view showing the indicated ChIP-seq and ATAC-seq datasets at a few example loci that undergo loss of accessibility in *Hox13*$^{-/-}$ distal E11.5 limb buds. **(g)** Heatmap showing wild type and *Hox13*$^{-/-}$ RNA expression values at distal enriched genes (from GSE81358). Rows are centered; unit variance scaling is applied to rows. Rows are clustered using Euclidean distance and average linkage. 361 rows, 4 columns. Source data are provided as a Source Data file.

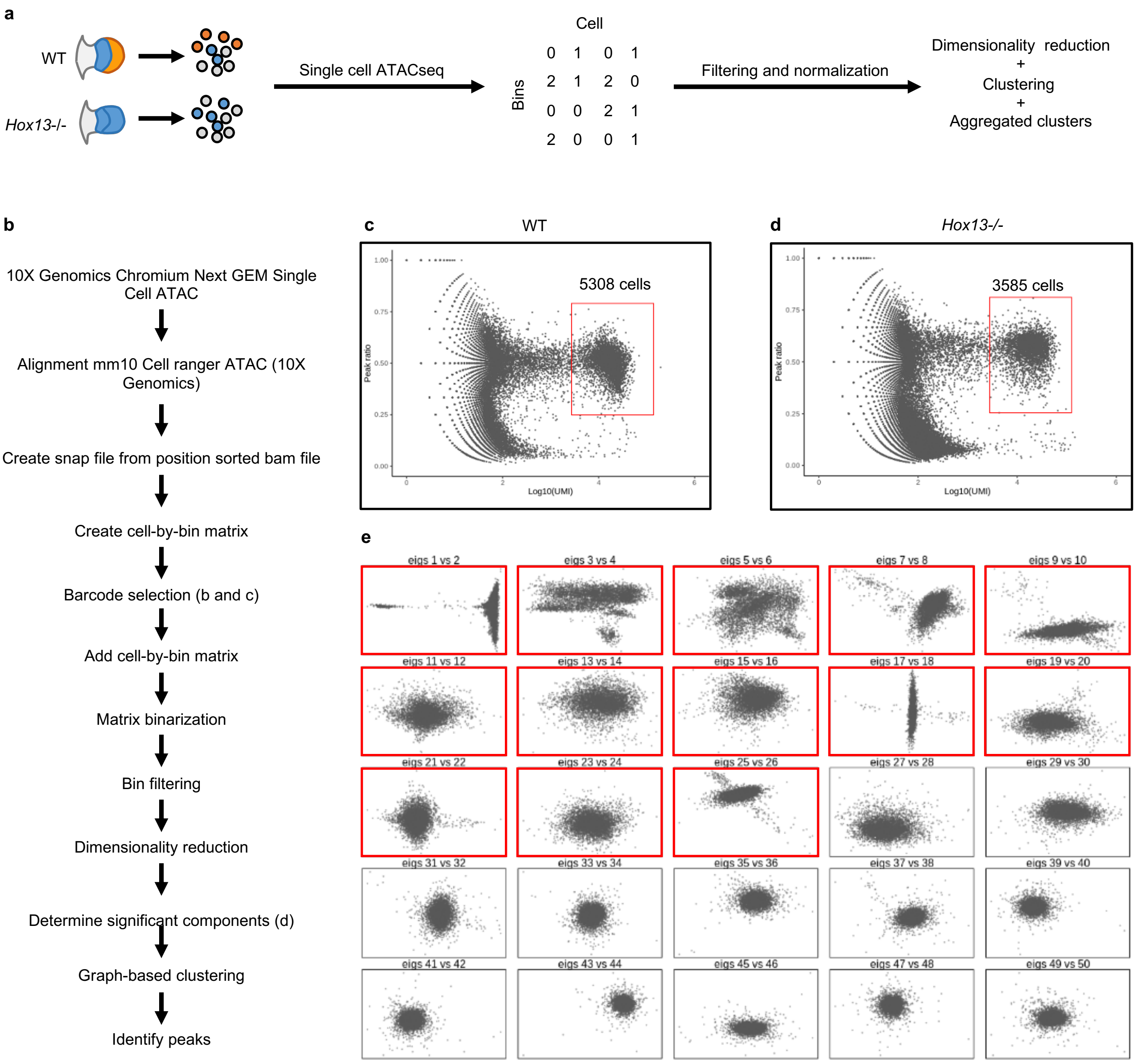

Supplementary Figure 6: Pipeline and quality control of scATACseq on WT and *Hox13-/-* limb bud

(**a**) Scheme of the single-cell ATAC-seq assay and data analysis performed. **(b)** Description of the pipeline used to analyze the single-cell ATAC-seq datasets generated in this study. **(c-d)** Dispersion plot of wild type **(c)** and *Hox13*$^{-/-}$ **(d)** showing the log10 (UMI number per each barcode) on the X axis and the fraction of reads in peaks per barcode on the Y axis. The red square shows the threshold that we used to consider barcodes as representing a captured cell. **(e)** Pairwise comparison of dimensions to determine the number of dimensions to use for dimensionality reduction (UMAP) and clustering of the scATAC-seq datasets.

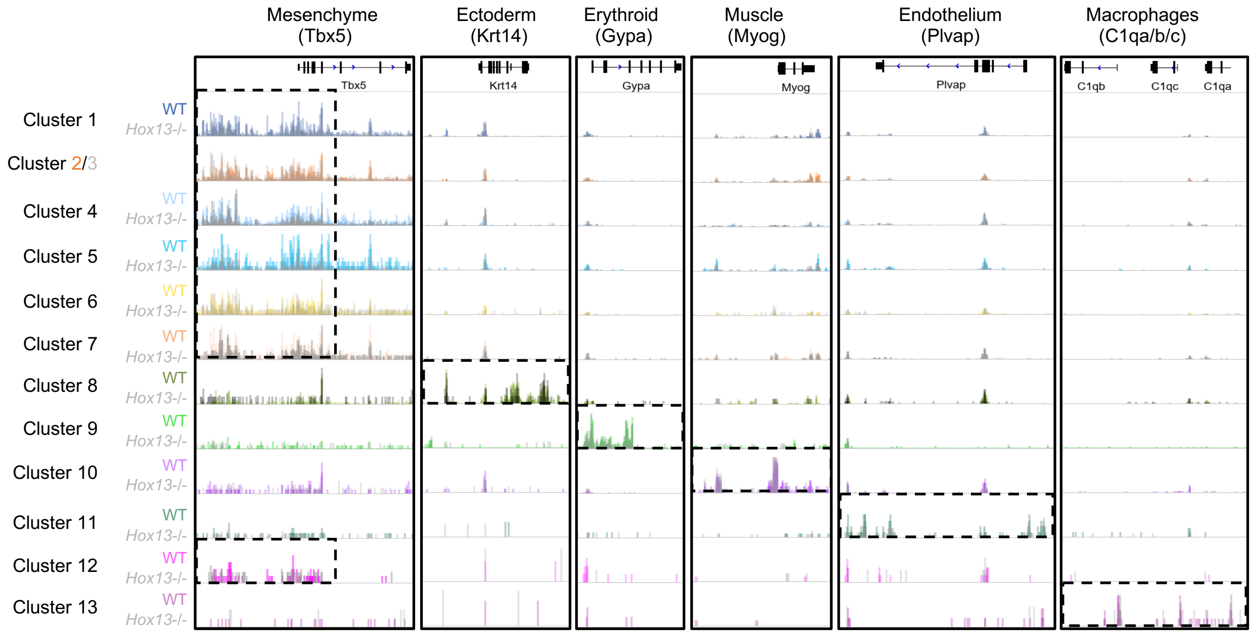

Supplementary Figure 7: Annotation of clusters identified by single cell ATAC-seq

Genome browser view (IGV) of aggregated signal of single cell ATAC-seq clusters separated by genotype (wild type, colored and *Hox13*$^{-/-}$, in grey) at marker loci for the indicated markers. Other markers were used to confirm correct identification (not shown).

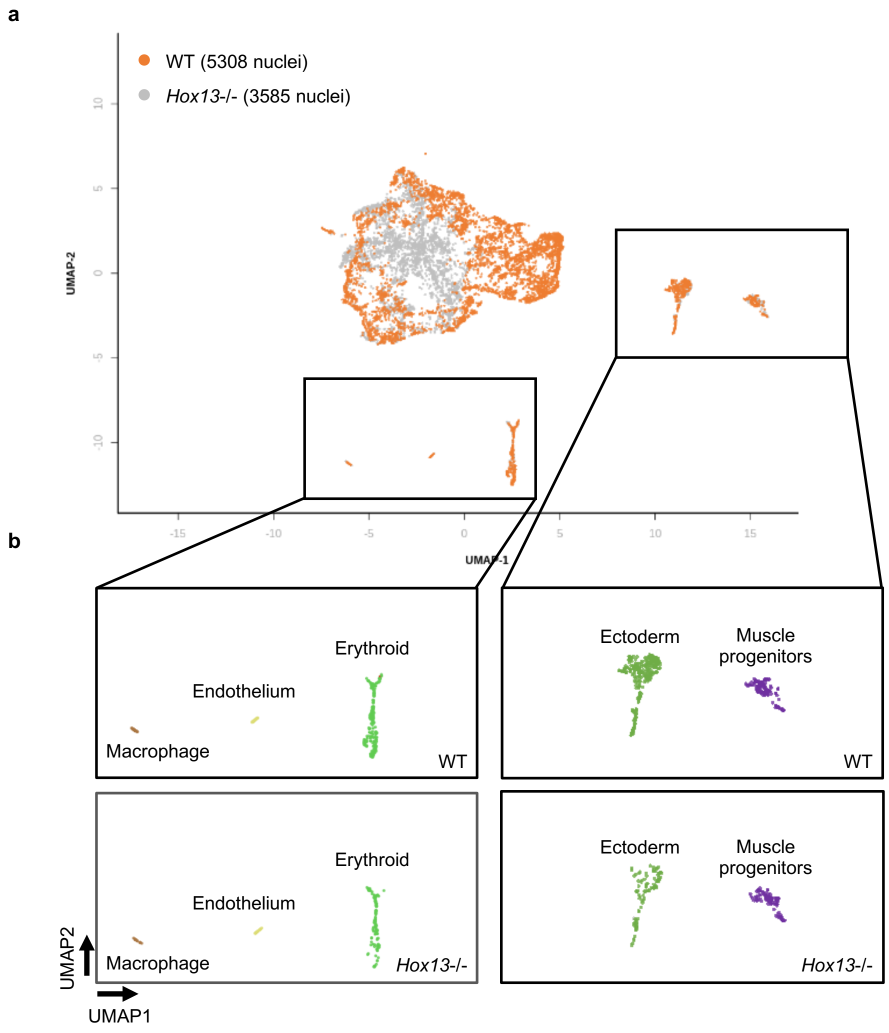

Supplementary Figure 8: Non-mesenchymal lineages are weakly affected by *Hox13* loss of function

(a) Dimensionality reduction map (UMAP) colored by genotype (wild type in orange, and *Hox13*$^{-/-}$ in grey). **(b)** Magnification of the squares from **(a)** separated by genotype (wild type, top and *Hox13*$^{-/-}$, bottom) showing the UMAP coordinates from the non-mesenchymal lineages.

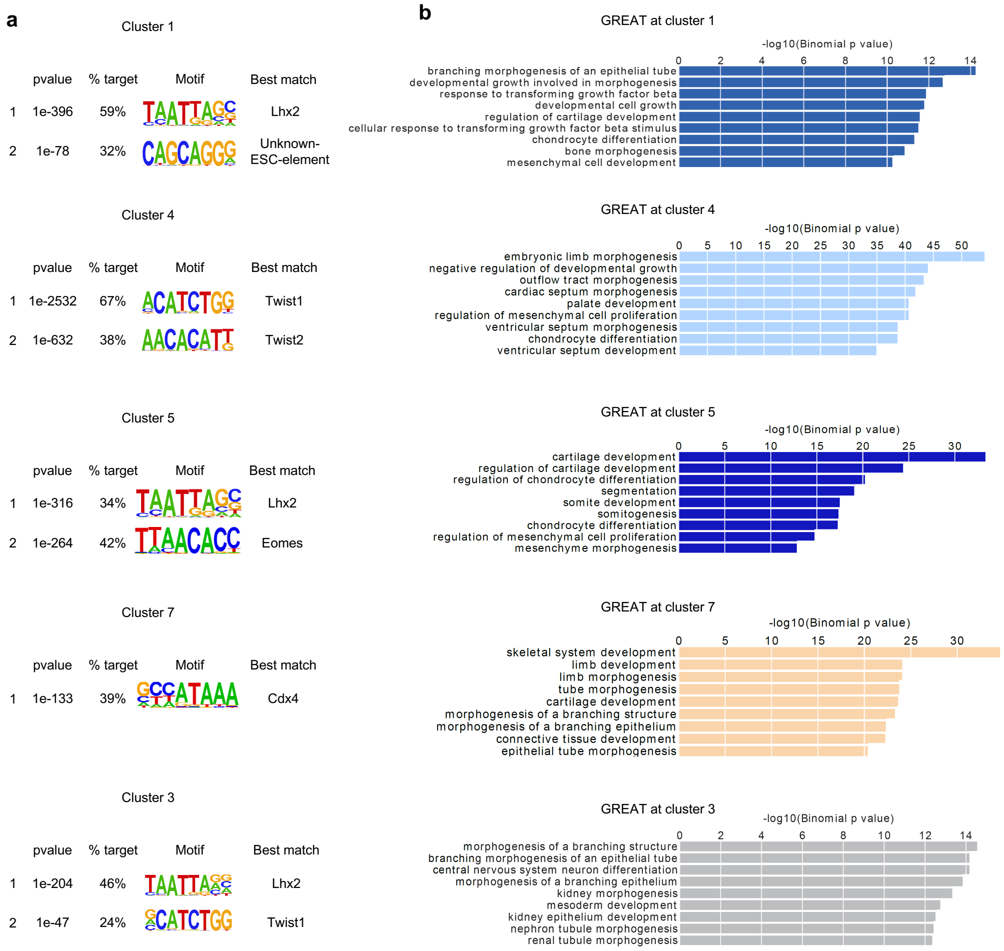

Supplementary Figure 9: Motifs and GREAT analysis on non distal limb scATAC clusters

(a) Top scoring motifs found by HOMER *de novo* motif analysis on the whole peak window at clusters 1, 4, 5, 7 and 3 peaks. **(b)** GREAT analysis showing the enriched GO terms (p-value computed using binomial test over genomic regions by GREAT) for biological processes for genes associated with clusters 1, 4, 5, 7 and 3.

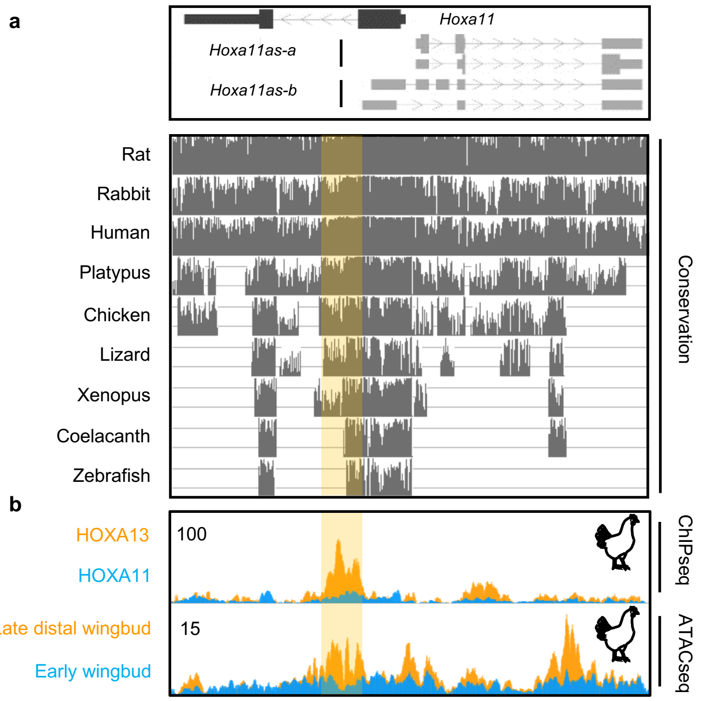

Supplementary Figure 10: Opening of the enhancer driving *Hoxa11* antisense transcription is conserved in Chicken

(a) Pairwise sequence conservation at the *Hoxa*11 locus. The HOX13-dependent enhancer driving antisense transcription in distal mouse limb bud is highlighted. **(b)** Genome browser view (IGV) of HOXA13 and HOXA11 ChIP-seq data (GSE86089) from primary chicken mesenchymal limb progenitor cells and ATAC-seq from chicken wing bud at early stage (HH20, blue), prior *Hox13* expression and late (HH26/27, orange) distal wing bud, in the *Hox13*-expressing domain at the *Hoxa11* locus.

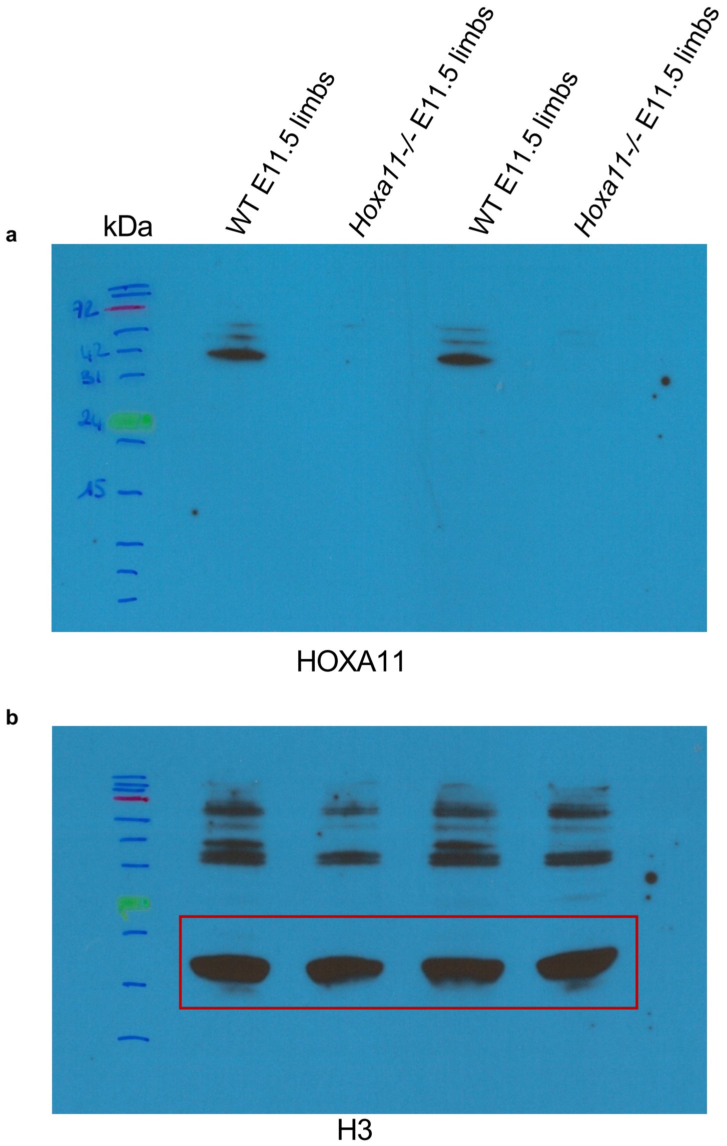

Supplementary Figure 11: Uncropped Original Scans

Uncropped original scans from Western blot of nuclear extracts from E11.5 wild type and *Hoxa11*$^{-/-}$ limb buds presented in supplementary figure 1. Two replicates are shown. Antibody against HOXA11 **(a)** and Histone H3 **(b)** were used.
