# Supplementary material for: HOX13-dependent chromatin accessibility underlies the transition towards the digit development program
Source: Nat Commun. 2020 May 19;11:2491. doi: 10.1038/s41467-020-16317-2 (PMC7237422; doi:10.1038/s41467-020-16317-2)
Supplement: Supplementary file 3 — Reporting Summary [file 41467_2020_16317_MOESM3_ESM.pdf]

nature research

Corresponding author(s): Dr. Marie Kmita

Last updated by author(s): Mar 31, 2020

Reporting Summary

Nature Research wishes to improve the reproducibility of the work that we publish. This form provides structure for consistency and transparency in reporting. For further information on Nature Research policies, see Authors & Referees and the Editorial Policy Checklist .

Statistics

For all statistical analyses, confirm that the following items are present in the figure legend, table legend, main text, or Methods section.

n/a	Confirmed	
☐	☒	The exact sample size (*n*) for each experimental group/condition, given as a discrete number and unit of measurement
☐	☒	A statement on whether measurements were taken from distinct samples or whether the same sample was measured repeatedly
☐	☒	The statistical test(s) used AND whether they are one- or two-sided<br>*Only common tests should be described solely by name; describe more complex techniques in the Methods section.*
☒	☐	A description of all covariates tested
☒	☐	A description of any assumptions or corrections, such as tests of normality and adjustment for multiple comparisons
☒	☐	A full description of the statistical parameters including central tendency (e.g. means) or other basic estimates (e.g. regression coefficient) AND variation (e.g. standard deviation) or associated estimates of uncertainty (e.g. confidence intervals)
☐	☒	For null hypothesis testing, the test statistic (e.g. *F*, *t*, *r*) with confidence intervals, effect sizes, degrees of freedom and *P* value noted<br>*Give P values as exact values whenever suitable.*
☒	☐	For Bayesian analysis, information on the choice of priors and Markov chain Monte Carlo settings
☐	☒	For hierarchical and complex designs, identification of the appropriate level for tests and full reporting of outcomes
☐	☒	Estimates of effect sizes (e.g. Cohen's *d*, Pearson's *r*), indicating how they were calculated

Our web collection on statistics for biologists contains articles on many of the points above.

Software and code

Policy information about availability of computer code

Data collection	The ChIP-seq data for HOX13 in distal limb and RNAseq for wild type and Hox13-/- distal limb buds at E11.5 are available from the NCBI Gene Expression Omnibus repository under the accession numbers GSE81356. Chicken ChIPseq data of Hoxa13 and Hoxa11 were obtained from the accession numbers GSE86088.
Data analysis	IGV software, DESeq2, Easeq1.02, HOMER v4.9.1, Samtools v1.4.1, MACS v2.1.1.20160309 and v2.1.2, Bowtie v.2.3.1, TopHat v2.1.0, Deseq2, Cellranger-atac v1.1.0, snaptools v1.4.1, SnapATAC v1.0.0, leidenalg v0.7.0, Bedtools 2.28.0, EdgeR v3.14.0, sratoolkit v2.8.2-1, R_Bioconductor v3.5.1_3.7, GREAT software.

For manuscripts utilizing custom algorithms or software that are central to the research but not yet described in published literature, software must be made available to editors/reviewers. We strongly encourage code deposition in a community repository (e.g. GitHub). See the Nature Research guidelines for submitting code & software for further information.

Data

Policy information about availability of data

All manuscripts must include a data availability statement. This statement should provide the following information, where applicable:

- Accession codes, unique identifiers, or web links for publicly available datasets
- A list of figures that have associated raw data
- A description of any restrictions on data availability

The ChIP-seq data for HOX13 in distal limb and RNA-seq for wild type and Hox13-/- distal limb buds at E11.5 are available from the NCBI Gene Expression Omnibus repository under the accession numbers GSE81356. Chicken ChIP-seq data of HOXA13 and HOXA11 were obtained from the accession numbers GSE86088. Genomic data will be deposited on Gene Expression Omnibus (GEO) before publication. All other relevant data supporting the key findings of this study are available within the article or from the corresponding author upon reasonable request.

Field-specific reporting

Please select the one below that is the best fit for your research. If you are not sure, read the appropriate sections before making your selection.

- [x] Life sciences
- [ ] Behavioural & social sciences
- [ ] Ecological, evolutionary & environmental sciences

For a reference copy of the document with all sections, see nature.com/documents/nr-reporting-summary-flat.pdf

Life sciences study design

All studies must disclose on these points even when the disclosure is negative.

Sample size	ChIP-seq were performed using a pool of an average of 60 forelimb buds (30 embryos), ATAC-seq were performed in duplicate from 50000 random cells from pooled left and right forelimb buds of a single embryo and scATAC-seq were performed using random cells from pooled left and right forelimb buds of a single embryo. No statistical methods were used to determine sample size. We chose the sample size based on literatures in the field.
Data exclusions	There was no exclusion/inclusion of samples or animals in the analysis
Replication	All attempts at replication were successful.<br>ATAC-seq and HOXA11 ChIP-seq in WT mice were done using two biological replicates<br>RNA-seq experiments were performed on three biological replicates<br>HOXA11 ChIP-seq in RA11KI mice was done using one replicate<br>HOXA11 ChIP-seq in Hox13-/- mice was done using one replicate<br>scATAC-seq in WT and Hox13-/- were done using one replicate
Randomization	There was no randomization of experiments. In this study we analyzed RNA-seq data, ChIP-seq data, ATAC-seq and scATAC-seq data with embryonic tissues.
Blinding	Investigators were not blinded during experiments and analysis because all samples were analyzed in the same way. Genotyping was done before performing the experiments.

Reporting for specific materials, systems and methods

We require information from authors about some types of materials, experimental systems and methods used in many studies. Here, indicate whether each material, system or method listed is relevant to your study. If you are not sure if a list item applies to your research, read the appropriate section before selecting a response.

Materials & experimental systems

n/a	Involved in the study	
[ ]	[x]	Antibodies
[x]	[ ]	Eukaryotic cell lines
[x]	[ ]	Palaeontology
[ ]	[x]	Animals and other organisms
[x]	[ ]	Human research participants
[x]	[ ]	Clinical data

Methods

n/a	Involved in the study	
[ ]	[x]	ChIP-seq
[x]	[ ]	Flow cytometry
[x]	[ ]	MRI-based neuroimaging

Antibodies

Antibodies used	HOXA11 (SAB1304728, Sigma)
Validation	In Supplementary Figure 1a, we validated the antibody by western-blot using WT and knockout mouse for HOXA11

Animals and other organisms

Policy information about studies involving animals; ARRIVE guidelines recommended for reporting animal research

Laboratory animals	Hoxa13null (Hoxa13Str), Hoxd13null (Hoxd13lacZ), RosaHoxa11 mouse lines were previously described. All mice were maintained in mixed background (C57BL/6 X 129). All mice were maintained in mixed background (C57BL/6 X 129). The mice were housed in a 12h light: dark cycle between 18°C and 26°C temperature with relative humidity of 30-70 percent and given ad-libitum access to food and water for the duration of the study. Noon of the day of the vaginal plug was considered as E0.5 and the experiments were done using e11.5 embryos.

Wild animals	This study did not involve wild animals
Field-collected samples	This study did not involve field-collected samples
Ethics oversight	Mice work at the Institut de Recherches Cliniques de Montréal (IRCM) was reviewed and approved by the IRCM animal care committee (protocols 2015-14 and 2017-10) in accordance with Canadian regulations. We have complied with all relevant ethical regulations.

Note that full information on the approval of the study protocol must also be provided in the manuscript.

ChIP-seq

Data deposition

- [x] Confirm that both raw and final processed data have been deposited in a public database such as GEO.
- [x] Confirm that you have deposited or provided access to graph files (e.g. BED files) for the called peaks.

Data access links<br>*May remain private before publication.*	Link to access data from ChIP-seq and ATAC-seq: https://www.ncbi.nlm.nih.gov/geo/query/acc.cgi?acc=GSE123482 - access code: mpsbamwivrybnkv<br>Link to access data from scATAC-seq: https://www.ncbi.nlm.nih.gov/geo/query/acc.cgi?acc=GSE145657 - access code: kvefoskidhubtwx
Files in database submission	Raw files and processed files of all ChIP-seq, ATAC-seq and scATAC-seq can be found on GEO under the accession number GSE123482 and GSE145657
Genome browser session<br>(e.g. UCSC)	BigWig files for visualization are provided on GEO under the accession number GSE123482 and GSE145657

Methodology

Replicates	ATAC-seq and HOXA11 ChIP-seq in WT mice were done using two biological replicates<br>HOXA11 ChIP-seq in RA11KI mice was done using one replicate<br>HOXA11 ChIP-seq in Hoxa31-/- mice was done using one replicate<br>scATAC-seq in WT and Hox13-/- were done using one replicate
Sequencing depth	HOXA11 ChIP-seq in WT rep1 has 101965670 total number of reads, 72987652 uniquely mapped reads and is paired-end<br>HOXA11 ChIP-seq in WT rep2 has 49518472 total number of reads, 48474259 uniquely mapped reads and is paired-end<br>HOXA11 ChIP-seq in RA11KI has 48474259 total number of reads, 31790952 uniquely mapped reads and is paired-end<br>HOXA11 ChIP-seq in Hox13-/- has 130570369 total number of reads, 89379062 uniquely mapped reads and is paired-end<br>ATAC-seq in proximal WT rep1 has 54934026 total number of reads, 33749203 uniquely mapped reads and is paired-end<br>ATAC-seq in proximal WT rep2 has 57507523 total number of reads, 37704269 uniquely mapped reads and is paired-end<br>ATAC-seq in distal WT rep1 has 61017754 total number of reads, 38578851 uniquely mapped reads and is paired-end<br>ATAC-seq in distal WT rep2 has 60436768 total number of reads, 34571856 uniquely mapped reads and is paired-end<br>ATAC-seq in distal Hox13-/- rep1 has 56294651 total number of reads, 34791774 uniquely mapped reads and is paired-end<br>ATAC-seq in distal Hox13-/- rep2 has 56259930 total number of reads, 35322840 uniquely mapped reads and is paired-end<br>scATAC-seq in WT has 143472318 total number of reads, 139753046 uniquely mapped reads and is paired-end<br>scATAC-seq in Hox13-/- has 133313344 total number of reads, 129402112 uniquely mapped reads and is paired-end
Antibodies	HOXA11 (SAB1304728, Sigma)
Peak calling parameters	Peaks were identified by comparing each sample to its input using MACS v2.1.1.20160309 callpeak function using the parameters: --bw 250 -g mm --mfold 10 30 -p 1e-5.<br>The script used for the scATAC-seq analysis can be found at this link: https://github.com/BCYasser/DigitMorphoScATAC/blob/master/analysis_script
Data quality	HOXA11 ChIP-seq in WT rep1 has 39869 total peaks with pvalue<0.00005; and 17436 peaks with a fold>5<br>HOXA11 ChIP-seq in WT rep2 has 36909 total peaks with pvalue<0.00005; and 26041 peaks with a fold>5<br>HOXA11 ChIP-seq in RA11KI has 55249 total peaks with pvalue<0.00005; and 39990 peaks with a fold>5<br>HOXA11 ChIP-seq in Hoxa31-/- has 75283 total peaks with pvalue<0.00005; 24557 with fold>5<br>ATAC-seq in proximal WT rep1 has 26734 total peaks with pvalue<0.00005; and 16081 peaks with a fold>5<br>ATAC-seq in proximal WT rep2 has 26198 total peaks with pvalue<0.00005; and 13610 peaks with a fold>5<br>ATAC-seq in distal WT rep1 has 42736 total peaks with pvalue<0.00005; and 25923 peaks with a fold>5<br>ATAC-seq in distal WT rep2 has 31313 total peaks with pvalue<0.00005; and 19747 peaks with a fold>5<br>ATAC-seq in distal Hoxa13-/- rep1 has 39116 total peaks with pvalue<0.00005; and 26701 peaks with a fold>5<br>ATAC-seq in distal Hoxa13-/- rep2 has 47127 total peaks with pvalue<0.00005; and 34199 peaks with a fold>5
Software	Easeq1.02, HOMER v4.9.1, MACS v2.1.1.20160309, Bowtie v.2.3.1, tophat v2.1.0, Deseq2, sratoolkit v2.8.2-1, R_Bioconductor v3.5.1_3.7<br>The script used for the scATAC-seq analysis can be found at this link: https://github.com/BCYasser/DigitMorphoScATAC/blob/master/analysis_script
